# Unraveling the Regulatory Impact of LncRNA Hnf1aos1 on Hepatic Homeostasis in Mice

**DOI:** 10.3390/ncrna11040052

**Published:** 2025-07-04

**Authors:** Beshoy Armanios, Jing Jin, Holly Kolmel, Ankit P. Laddha, Neha Mishra, Jose E. Manautou, Xiao-Bo Zhong

**Affiliations:** 1 Department of Pharmaceutical Sciences, School of Pharmacy, University of Connecticut, Storrs, CT 06269, USA; beshoy.armanios@uconn.edu (B.A.); jingjin.w@uconn.edu (J.J.); holly.kolmel@uconn.edu (H.K.); apladdha@uconn.edu (A.P.L.); jose.manautou@uconn.edu (J.E.M.); 2 Department of Pathobiology, University of Connecticut, Storrs, CT 06269, USA; neha.mishra@uconn.edu

**Keywords:** lipid metabolism, liver inflammation, hepatotoxicity, bile acid synthesis, lncRNA Hnf1aos1

## Abstract

**Background/Objectives:** Long non-coding RNAs (lncRNAs) play significant roles in tissue development and disease progression and have emerged as crucial regulators of gene expression. The hepatocyte nuclear factor alpha antisense RNA 1 (HNF1A-AS1) lncRNA is a particularly intriguing regulatory molecule in liver biology that is involved in the regulation of cytochrome P450 enzymes via epigenetic mechanisms. Despite the growing recognition of lncRNAs in liver disease, the comprehensive role of HNF1A-AS1 in liver function remains unclear. This study aimed to investigate the roles of the mouse homolog of the human HNF1A-AS1 lncRNA HNF1A opposite strand 1 (Hnf1aos1) in liver function, gene expression, and cellular processes using a mouse model to identify potential therapeutic targets for liver disorders. **Methods:** The knockdown of Hnf1aos1 was performed in in vitro mouse liver cell lines using siRNA and in vivo livers of AAV-shRNA complexes. Changes in the global expression landscapes of mRNA and proteins were revealed using RNA-seq and proteomics, respectively. Changes in the selected genes were further validated via real-time quantitative polymerase chain reaction (RT-qPCR). Phenotypic changes were assessed via histological and absorbance-based assays. **Results:** After the knockdown of Hnf1aos1, RNA-seq and proteomics analysis revealed the differential gene expression of the mRNAs and proteins involved in the processes of molecular transport, liver regeneration, and immune signaling pathways. The downregulation of ABCA1 and SREBF1 indicates their role in cholesterol transport and fatty acid and triglyceride synthesis. Additionally, significant reductions in hepatic triglyceride levels were observed in the Hnf1aos1-knockdown group, underscoring the impact on lipid regulation. Notably, the knockdown of Hnf1aos1 also led to an almost complete depletion of CYP7A1, the rate-limiting enzyme in bile acid synthesis, highlighting its role in cholesterol homeostasis and hepatotoxicity. Histological assessments confirmed these molecular findings, with increased hepatic inflammation, hepatocyte swelling, and disrupted liver architecture observed in the Hnf1aos1-knockdown mice. **Conclusions:** This study illustrated that Hnf1aos1 is a critical regulator of liver health, influencing both lipid metabolism and immune pathways. It maintains hepatic lipid homeostasis, modulates lipid-induced inflammatory responses, and contributes to viral immunity, indirectly affecting glucose and lipid metabolic balance.

## 1. Introduction

Long non-coding RNAs (lncRNAs) have emerged as crucial regulators of gene expression and play significant roles in tissue development, homeostasis, and disease progression. Despite their importance, the role of lncRNAs in regulating sophisticated biological processes, particularly those in the liver, remains largely unexplored. This gap in knowledge underscores the need for comprehensive studies using advanced models to understand the intricate regulatory networks involving lncRNAs and their partners. Several lncRNAs have been implicated in liver diseases, particularly in hepatocellular carcinoma (HCC). MALAT1, NEAT1, HEIH, HULC, HOTTIP, HOTAIR, and DANCR/ANCR function as oncogenic lncRNAs in HCC, promoting cancer progression through mechanisms such as activating oncogenic splicing factors, sponging tumor suppressor miRNAs, and altering gene expression patterns [1,2]. Conversely, MEG3, FENDRR, DILC, and GAS5 act as tumor suppressors in HCC by regulating miRNA expression, modulating signaling pathways, and downregulating tumor suppressor genes. Some lncRNAs, like H19, have both oncogenic and tumor-suppressive roles in HCC [1]. DANCR/ANCR have been identified as oncogenic lncRNAs in cholangiocarcinoma [1]. Understanding these roles and mechanisms will provide valuable insights into the potential diagnostic biomarkers and therapeutic targets for hepatic disorders.

A critical challenge in lncRNA research is the limited conservation of most lncRNAs between species. Unlike protein-coding genes, which are normally conserved, the majority of lncRNAs exhibit poor sequence conservation across species, with only a small fraction of lncRNAs showing detectable homology between humans and mice [3,4]. For example, only about 6–19% of antisense lncRNAs are conserved between humans and mice, and the conservation rate is even lower for non-overlapping lncRNAs [5]. This low conservation rate complicates the translation of lncRNA biology from model organisms to humans and raises concerns about the functional relevance of the findings obtained in animal models [6]. Furthermore, even when sequence or positional conservation exists, lncRNAs may be processed differently or have divergent functions across species due to differences in subcellular localization and interacting partners.

Nevertheless, the subset of lncRNAs that are conserved between humans and mice tend to have longer gene bodies, more exons, higher expression levels, and stronger associations with human diseases, indicating their potential functional importance. These conserved lncRNAs are more likely to play critical roles in fundamental biological processes and are thus particularly valuable for mechanistic studies using animal models.

Importantly, hepatocyte nuclear factor 1 alpha (HNF1A) antisense RNA 1 (HNF1A-AS1) and its mouse ortholog, Hnf1a opposite RNA 1 (Hnf1aos1), are among the minority of lncRNAs that are conserved between species. This conservation is characterized not only by their positional relationship, both being transcribed from the antisense strand of the *HNF1A* gene locus within intron 1, but also by the preservation of regulatory elements, such as conserved HNF1A binding sites and nucleosome-free regions in their promoters [7]. Although the overall RNA sequence similarity between human HNF1A-AS1 and mouse Hnf1aos1 is relatively low (less than 60%), their genomic positioning and regulatory architectures are evolutionarily preserved. This positional and regulatory conservation is functionally significant: both lncRNAs participate in bidirectional feedback loops with HNF1A, where HNF1A activates their transcription, and in turn, the lncRNAs stabilize HNF1A mRNA and protein levels through mechanisms such as chromatin insulation and protection from ubiquitin-mediated degradation [7,8,9,10,11,12,13].

Functionally, both HNF1A-AS1 in humans and Hnf1aos1 in mice regulate key aspects of liver biology, including hepatocyte differentiation, drug metabolism, and responses to cellular stress. Both interact with the Polycomb Repressive Complex 2 (PRC2) via EZH2, implicating them in conserved epigenetic regulatory pathways [14,15]. The functional conservation of these lncRNAs, despite limited sequence homology, highlights the importance of studying them in mouse models to gain insights relevant to human liver biology and diseases.

The liver-enriched transcription factor HNF1A and its antisense lncRNA, HNF1A-AS1, represent a particularly intriguing regulatory pair in liver biology. HNF1A regulates HNF1A-AS1 by directly binding to seven recognition sites in the *HNF1A-AS1* promoter, thereby driving its transcription in pancreatic and hepatic cells [7,16]. Recent studies have clarified that the regulatory influence of HNF1A-AS1 on HNF1A transcription is mediated primarily by its DNA sequence elements within the promoter, rather than by the HNF1A-AS1 RNA transcripts themselves. Specifically, the promoter region of *HNF1A-AS1* modulates chromatin 3D interactions and insulates the *HNF1A* promoter from intronic enhancers, thereby preventing aberrant activation [7,16]. In contrast, the HNF1A-AS1 RNA transcript can bind to HNF1A protein, blocking TRIM25-mediated ubiquitination and proteasomal degradation, and thus stabilizing HNF1A at the protein level [13]. These findings highlight that HNF1A-AS1 exerts its regulatory effects through both cis-acting DNA elements and trans-acting RNA mechanisms, depending on the biological context. Therefore, genetic ablation of the *HNF1A-AS1* promoter disrupts chromatin insulation and reduces HNF1A transcription independent of RNA transcript levels, while the knockdown of HNF1A-AS1 RNA affects HNF1A protein stability. HNF1A is essential for hepatocyte differentiation, drug metabolism, and liver functions [7,10,17]. Studies have shown that HNF1A expression is significantly repressed in fibrotic liver tissues, and that its knockdown exacerbates hepatic fibrogenesis [10,11]. Moreover, *Hnf1a* knockout mice exhibited enlarged livers, progressive liver damage, and the spontaneous development of hepatocellular carcinoma through fatty liver without cirrhosis [18]. A recent study also identified several HNF1A-AS1 transcripts in liver samples and cell lines, with expression patterns highly correlated with hepatocyte differentiation and various liver disease conditions [19].

Similarly to HNF1A-AS1, the mouse ortholog lncRNA Hnf1aos1 functions as a regulator of HNF1A. In the liver, Hnf1aos1 acts as a transcriptional stabilizer of HNF1A, forming a feedback loop that controls HNF1A function. Hnf1aos1 is involved in the regulation of cytochrome P450 (CYP) enzymes and contributes to susceptibility to acetaminophen-induced cytotoxicity in liver cells [20]. It also plays a role in promoting hepatocellular carcinoma cell proliferation [21]. Furthermore, Hnf1aos1 interacts with enhancer of zeste homolog 2 (EZH2), the catalytic subunit of Polycomb Repressive Complex 2 (PRC2), in liver tissue, suggesting its involvement in epigenetic regulation [22]. The complex functions of Hnf1aos1 in liver metabolism, drug response, and cancer progression make it a potential target for therapeutic interventions in various liver disorders. However, the specific regulatory mechanisms of Hnf1aos1 and its effect on liver function remain largely unexplored. While some studies have identified Hnf1aos1’s involvement in hepatic gene expression and metabolism, the full extent of its influence on liver homeostasis, development, and disease progression is yet to be fully understood [20].

These examples highlight the potential of antisense lncRNAs in modulating the expression and function of critical regulatory proteins. Understanding the functions of Hnf1aos1 in liver development and disease is essential, as it could lead to novel therapeutic strategies for liver disorders and potentially other severe or untreatable diseases. Previous studies have shown that acetaminophen-induced cytotoxicity in liver cells is associated with low HNF1A-AS1 expression, suggesting its potential as a therapeutic target [8]. Furthermore, HNF1A-AS1 has been implicated in tumor development and its expression is dysregulated in numerous cancers [12,23,24,25].

Given the critical role of HNF1A in liver function and the emerging importance of lncRNAs in gene regulation, we hypothesized that the knockdown of Hnf1aos1 causes significant liver dysregulation. This study aims to gain a broader understanding of the specific effects of Hnf1aos1 knockdown on liver function, gene expression patterns, and cellular processes in a mouse model. By investigating this hypothesis, we sought to uncover new insights into the regulatory mechanisms governing liver homeostasis and identify novel therapeutic targets for liver diseases.

## 2. Results

### 2.1. Effective Knockdown of lncRNA Hnf1aos1 in In Vitro Hepa1C1c7 Cells via siRNA and In Vivo Mouse Liver via rAAV8-sh-Hnf1aos1 Approaches

**Figure 1:**
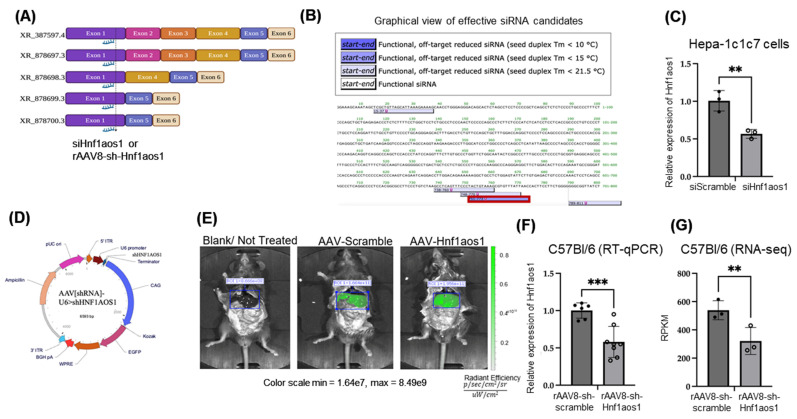
Knockdown of Hnf1aos1 in in vitro and in vivo models. (**A**) Strategy for Hnf1aos1 knockdown using siRNA in Hepa1c1c7 cells (in vitro) and rAAV8-shRNA in C57BL/6 mice (in vivo), targeting all annotated Hnf1aos1 transcripts. Schematic representation of effective siRNA candidates targeting exon 1 of Hnf1aos1. (**B**) Schematic representation of effective siRNA candidates targeting exon 1 of Hnf1aos1. (**C**) qPCR analysis demonstrating significant reduction in Hnf1aos1 RNA levels following siHnf1aos1 treatment in Hepa1c1c7 cells. (**D**) Schematic of the pAAV[shRNA]-U6 > Hnf1aos1 plasmid construct used for in vivo shRNA-mediated knockdown of Hnf1aos1, featuring a U6 promoter-driven shRNA, rAAV8 capsid for liver targeting, and a CAG promoter-driven EGFP for tracking transduction efficiency. (**E**) In vivo imaging of EGFP expression in control, rAAV8-scramble, and rAAV8-Hnf1aos1-shRNA-injected mice. Radiant efficiency ([photons/sec/cm^2^/sr]/[μW/cm^2^]) quantifies EGFP signal intensity, validating successful AAV-mediated transduction. (**F**) qPCR analysis showing significant reduction in Hnf1aos1 RNA levels following rAAV8-Hnf1aos1 treatment in vivo. (**G**) RNA-seq confirmation of Hnf1aos1 knockdown efficiency. Data are presented as mean ± SD. Statistical significance was determined by two-tailed unpaired *t*-test; ** *p* < 0.01 and *** *p* < 0.001.

To ensure the effective knockdown of all Hnf1aos1 transcript variants, we designed siRNA-targeting exon 1, which is present in all five annotated alternative transcripts of the mouse *Hnf1aos1* gene (Figure 1A). The target sequence was selected based on its minimal off-target effects and strong seed duplex stability, optimizing both specificity and efficacy in reducing Hnf1aos1 expression, as predicted by siDirect, a tool used to design highly effective and target-specific siRNA for gene silencing (Figure 1B) (https://sidirect2.rnai.jp/, accessed on 26 September 2023). The knockdown efficacy of the designed siRNA was first tested in an in vitro model of the mouse Hepa1c1c7 cell line (Figure 1C), which exhibited a significant reduction of 50% in Hnf1aos1 RNA levels, as confirmed via RT-qPCR analysis, with a scrambled siRNA sequence as a control that did not target any mouse genomic sequence.

To effectively knockdown all possible Hnf1aos1 transcripts in the mouse liver, an adeno-associated virus vector plasmid containing the sh-Hnf1aos1 sequence (pAAV[shRNA]-U6 > sh-Hnf1aos1) was designed (Figure 1D), which contains the U6 promoter to drive sh-Hnf1aos1 expression. To monitor transfection efficiency in the mouse liver, the construct also included an enhanced green fluorescent protein (EGFP) reporter, allowing successful delivery to be visualized by green fluorescence. The in vivo imaging system (IVIS) revealed successful transduction in both the rAAV8-sh-scramble and rAAV8-sh-Hnf1aos1 groups, indicating robust EGFP expression only in the liver with pAAV transfection, compared to that in the blank mouse without pAAV transfection (Figure 1E).

The expression of Hnf1aos1 RNA levels in the liver tissues of mice treated with rAAV8-sh- Hnf1aos1 and rAAV8-sh-scramble was quantified via RT-qPCR and showed a 40% reduction in Hnf1aos1 RNA levels in the rAAV8-sh-Hnf1aos1 group (*n* = 8) compared to the rAAV8-scramble control group (*n* = 6) (Figure 1F), consistent with the knockdown efficacy in the in vitro knockdown results (Figure 1C). Statistical analysis using an unpaired two-tailed *t*-test confirmed that the observed knockdown was significant (*** *p* < 0.001), validating the effectiveness of both siRNA and rAAV8-shRNA approaches in targeting Hnf1aos1. These results highlight the efficacy of this knockdown strategy, which leverages the same siRNA target sequence for both in vitro and in vivo applications.

### 2.2. The Knockdown of Hnf1aos1 Induces Widespread Transcriptional Changes in Liver Metabolism and Immune Pathways

**Figure 2:**
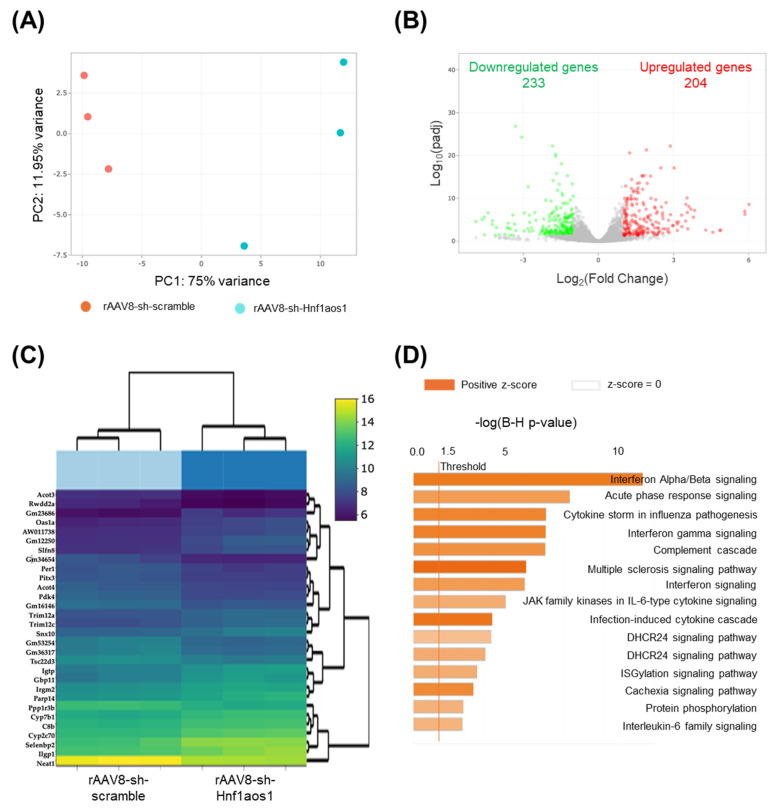
Comprehensive analysis of differential gene expression and functional enrichment following RNA-seq. (**A**) Samples were projected onto a 2D plane spanned by their first two principal components (PC) (*x*: PC1 and *y*: PC2). (**B**) Volcano plot depicting global gene expression changes. Red and green dots represent significantly upregulated (adjusted *p* < 0.05, log_2_(fold change) > 1) and downregulated (adjusted *p* < 0.05, log_2_(fold change) < −1) genes, respectively. The −log_10_(padj) refers to the negative base-10 logarithm of the adjusted *p*-value. (**C**) A hierarchical clustering heatmap of top 30 differentially expressed genes in Hnf1aos1 knockdown versus the scramble control mice. (**D**) Gene Ontology (GO) enrichment analysis shows significantly impacted signaling pathways, ranked via −log(B-H *p*-value). Orange and blue bars indicate upregulated and downregulated pathways in Hnf1aos1 knockdown mice, respectively. The orange vertical line denotes the significance threshold at −log(B-H *p*-value) = 1.3, equivalent to *p* = 0.05.

An RNA-seq approach was used to reveal transcriptomic alterations in both mRNAs and lncRNAs between the AVV8-scramble (*n* = 3) and rAAV8-sh-Hnf1aos1 groups (*n* = 3). RNA-seq data were analyzed using the DESeq2 package, which normalized raw read counts by adjusting for sequencing depth and sample composition using the median-of-ratios method. This approach is considered the gold standard for differential expression analysis and enables a robust comparison of gene expression between experimental groups. Normalized counts for Hnf1aos1 are presented, confirming efficient knockdown in the experimental group (Figure 1G). Principal component analysis (PCA) was conducted to visualize the overall transcriptional variances between the sh-scramble control (red dots) and sh-Hnf1aos1 knockdown samples (blue dots), as well as the inter-individual variability within each group (Figure 2A). PCA illustrates a clear differentiation between the scramble and knockdown groups, with smaller variability within the groups. The volcano plot depicts the changes in global gene expression, with 233 red dots representing upregulated genes (adjusted *p* < 0.05, log_2_(fold change) > 1) and 204 green dots representing downregulated genes (adjusted *p* < 0.05, log_2_(fold change) < −1) (Figure 2B). Hierarchical clustering of the top 30 differentially expressed genes is shown in Figure 2C, including some genes with functions involved in immune response and lipid metabolism, with distinct expression profiles between the groups. Gene Ontology (GO) enrichment analysis highlighted multiple altered signaling pathways, ranked via −log(B-H *p*-value), revealing significant changes across several biological processes, particularly those associated with metabolism, inflammatory response, fibrosis, and cellular stress (Figure 2D). Comprehensive analysis of differential gene expression and functional enrichment following RNA-seq revealed significant alterations in various metabolic- and immune-related pathways following Hnf1aos1 knockdown.

**Figure 3:**
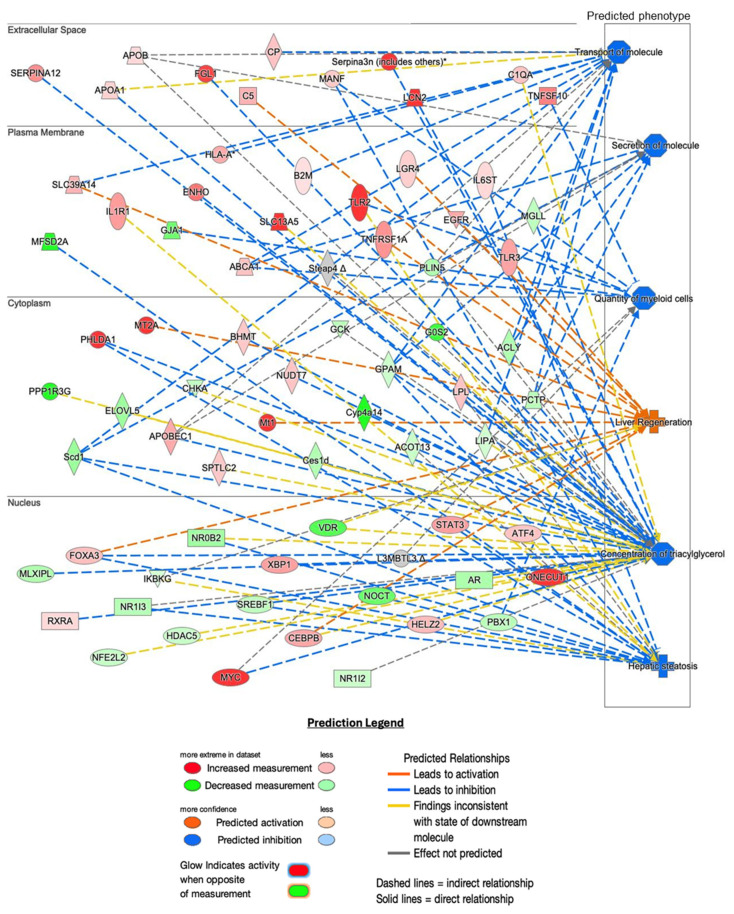
Pathway analysis of RNA-seq data. Predicted downstream physiological changes from differentially expressed genes were analyzed using Qiagen IPA software 24.0.2. The panel presents predicted diseases/changes (z-score > 1.99) and the top genes in the dataset affecting them (z-score > 1.99). * indicates other members of the Serpina family.

Ingenuity Pathway Analysis was used to map differentially expressed genes, which revealed a complex network of interactions linking Hnf1aos1 knockdown to downstream physiological changes (Figure 3). Network analysis of liver tissue mRNA expression predicted significant effects on liver health and metabolism. This analysis revealed shifts in the cellular phenotypes and processes related to the transport of molecules, secretion, myeloid cells, and lipid homeostasis. The dysregulation of these processes occurs alongside the differential expression of inflammatory (*Tlr2*, *Tnfrsf1a*, and *Serpina3n*) and lipid metabolism genes (*Scd* and *Cyp4a14*) [26,27,28,29,30]. Network analysis of mRNA expression predicted several pathways. The specific predicted phenotypes included (1) the downregulation of both the passive and active transport of molecules, (2) decreased secretion of molecules, (3) decreased quantity of myeloid cells, (4) increased liver regeneration, (5) decreased hepatic concentration of triacylglycerols, and (6) increased hepatic steatosis. This combination indicates a possible shift toward cellular stress, with a regulatory imbalance in lipid regulation.

**Figure 4:**
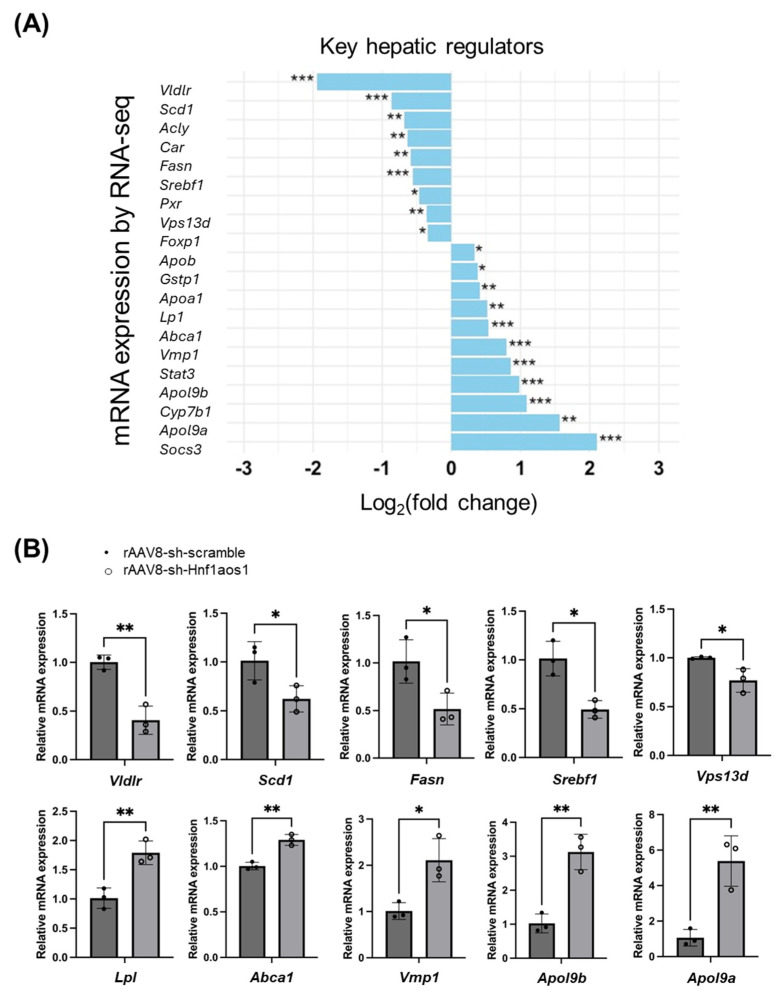
Key hepatic regulators and differential gene expression analysis. (**A**) Bar graph depicting the log_2_(fold change) in the expression of key hepatic regulatory genes following treatment with rAAV8-Hnf1aos1 compared to rAAV8-scramble, as determined by RNA-seq. Positive fold changes indicate upregulation, while negative fold changes indicate downregulation. (**B**) Confirmation of RNA-seq findings via real-time quantitative PCR (RT-qPCR). Relative mRNA expression levels of selected genes (*Fasn*, *Lpl*, *Shp-1*, *ApoI9a*, *ApoI9b*, *Abca1*, *Vmp1*, *Vps13d*, *Vldlr*, and *Srebf1*) are shown between rAAV8-Hnf1aos1 and rAAV8-scramble (*n* = 3). The data are represented as mean ± SD. Statistical significance is indicated as * *p* < 0.05, ** *p* < 0.01, or *** *p* < 0.001.

Based on the identification of major hepatic regulators that were significantly dysreg-ulated in the RNA-seq analysis (Figure 4A), quantitative PCR (qPCR) was employed to validate the expression changes of selected key genes. The qPCR results confirmed the dysregulation of these hepatic regulators, including Vldlr, Scd1, Fasn, Srebf1, and others, demonstrating consistent directional changes between the RNA-seq and qPCR datasets (Figure 4B).

We also evaluated the expression of *Oas* family genes using RNA-seq data, since *Oasl1* and *Oasl2* are positioned directly upstream (in cis) of *Hnf1aos1* in the mouse genome. Several members of the *Oas* family were significantly upregulated (Supplemental Figure S1), consistent with their well-established roles in viral immunity [31,32,33]. Specifically, *Oas1a* showed a notable log_2_ fold change of 2.49, while *Oasl1* and *Oasl2* displayed strong upregulation, with log_2_ fold changes of 2.70 and 2.41, respectively. *Oas1g* and *Oas1c* followed a similar trend, with log_2_ fold changes of 2.27 and 1.74, respectively [34]. *Oas3* was significantly upregulated, with a log_2_ fold change of 1.22, further supporting its role in antiviral defense. *Oas1b* demonstrated moderate upregulation (log_2_(fold change): 1.07), whereas *Oas2* showed a smaller, non-significant increase (log_2_(fold change): 0.61), suggesting a potential, but less pronounced contribution to viral immunity.

Collectively, these results demonstrated that Hnf1aos1 knockdown induces widespread transcriptomic changes, affecting genes involved in lipid metabolism, bile acid synthesis, drug metabolism, and inflammatory responses. These alterations suggest the crucial role of Hnf1aos1 in maintaining liver metabolic homeostasis and modulating immune function.

### 2.3. Hnf1aos1 Knockdown Induces Widespread Proteomic Changes in Liver Metabolism and Immune Pathways

Proteomic analysis was performed to investigate the effect of Hnf1aos1 knockdown on the liver tissue. The results revealed significant alterations in protein expression, providing insights into the biological processes affected by this lncRNA.

**Figure 5:**
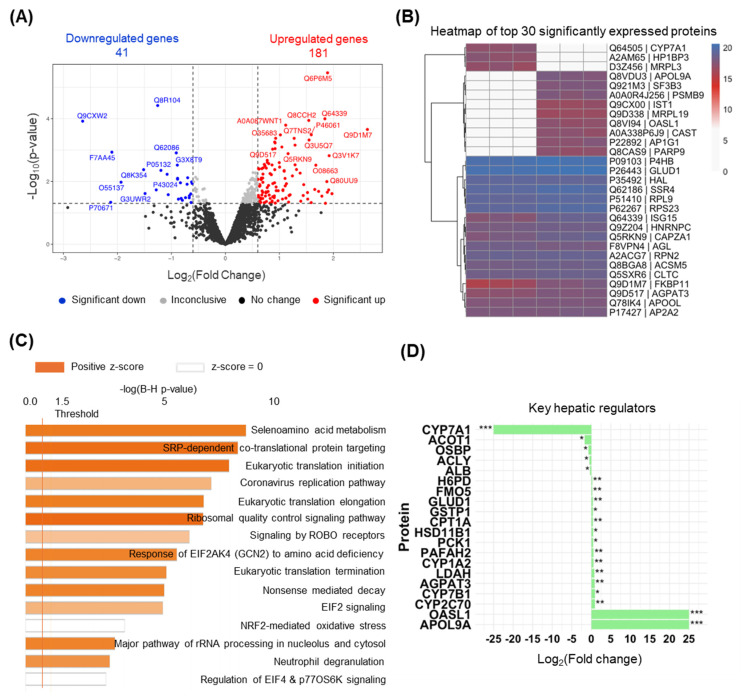
Differential protein expression and functional enrichment following proteomic analysis. (**A**) Volcano plot depicting global proteomic changes between Hnf1aos1 knockdown and scramble groups. Each point represents a protein with upregulated proteins (red dots; adjusted *p* < 0.05, log_2_ fold change > 0.6) and downregulated proteins (blue dots; adjusted *p* < 0.05, log_2_ change < −1) highlighted. (**B**) Hierarchical clustering and heatmap of top 30 differentially expressed proteins between Hnf1aos1 knockdown and scramble control mice. (**C**) Gene Ontology (GO) enrichment analysis identifies significantly impacted signaling pathways. The *y*-axis ranks pathways, while the *x*-axis shows the −log(B-H *p*-value). Orange bars indicate upregulated pathways (positive z-scores) in the Hnf1aos1 knockdown group; blue bars represent downregulated pathways (negative z-scores). The orange vertical line at 1.3 marks the significance threshold −log(B-H *p*-value) of 0.05. (**D**) Differentially expressed proteins involved in lipid metabolism, nuclear receptor signaling, and liver functions, as measured via proteomics. Statistical significance is indicated as * *p* < 0.05, ** *p* < 0.01, or *** *p* < 0.001.

A volcano plot illustrates the proteomic changes observed between the Hnf1aos1 knockdown and scramble control groups (Figure 5A). The plot highlights the distinct protein populations with altered expression levels. Specifically, 181 proteins were significantly upregulated (log_2_(fold change) > 0.6, adjusted *p* < 0.05, red dots), and 41 proteins were significantly downregulated (log_2_(fold change) < −0.6, adjusted *p* < 0.05, blue dots). This visualization underscores the magnitude and directionality of the proteomic changes induced by Hnf1aos1 knockdown.

Hierarchical clustering and heatmap analysis of the top 30 significantly differentially expressed proteins confirmed distinct expression profiles between the experimental groups (Figure 5B), demonstrating that Hnf1aos1 knockdown affects liver protein homeostasis. The heatmap visually represents clear segregation between groups based on protein expression patterns.

Pathway analysis using Qiagen IPA software further elucidated the functional consequences of Hnf1aos1 knockdown. GO enrichment analysis identified several significantly affected pathways (Figure 5C). Upregulated pathways (positive z-scores, orange bars) include signal recognition particle (SRP)-dependent co-translational proteins targeting the membrane, eukaryotic translation initiation, and selenoamino acid metabolism. These pathways suggest disruptions in protein synthesis and membrane targeting in response to Hnf1aos1 knockdown. The *x*-axis displays the −log(B-H *p*-value), indicating the significance of pathway enrichment.

Differential protein expression analysis revealed notable changes in key hepatic regulators following Hnf1aos1 knockdown, consistent with transcriptomic findings (Figure 5D). Among the most significantly altered proteins, CYP7A1 was dramatically downregulated (log_2_(fold change): −15.09), suggesting impaired bile acid synthesis, which is a critical pathway in hepatic metabolism. Conversely, APO19A and CYP7B1 were substantially upregulated (log_2_ fold changes: 25 and 0.93, respectively), indicating potential compensatory mechanisms for cholesterol efflux (from the liver to the bloodstream) and bile acid metabolism. The proteins involved in lipid metabolism showed varied responses: ACLY (log_2_ fold change: −0.51) was downregulated, indicating reduced cytosolic acetyl-CoA production, whereas CPT1A (log_2_ fold change: 0.38) was slightly upregulated, suggesting increased fatty acid oxidation. The proteins associated with detoxification and oxidative stress, such as GSTP1 (log_2_ fold change: 0.38), were upregulated, highlighting their enhanced protective mechanisms against cellular stress. Additionally, the proteins involved in gluconeogenesis (PCK1, log_2_ fold-change: 0.49) and lipid remodeling (LDAH, log_2_ fold-change: 0.77) were moderately upregulated, reflecting shifts in hepatic energy homeostasis. The significant upregulation of HSD11B1 (log_2_ fold change: 0.49), a regulator of glucocorticoid metabolism, suggests potential alterations in the inflammatory signaling pathways.

**Figure 6:**
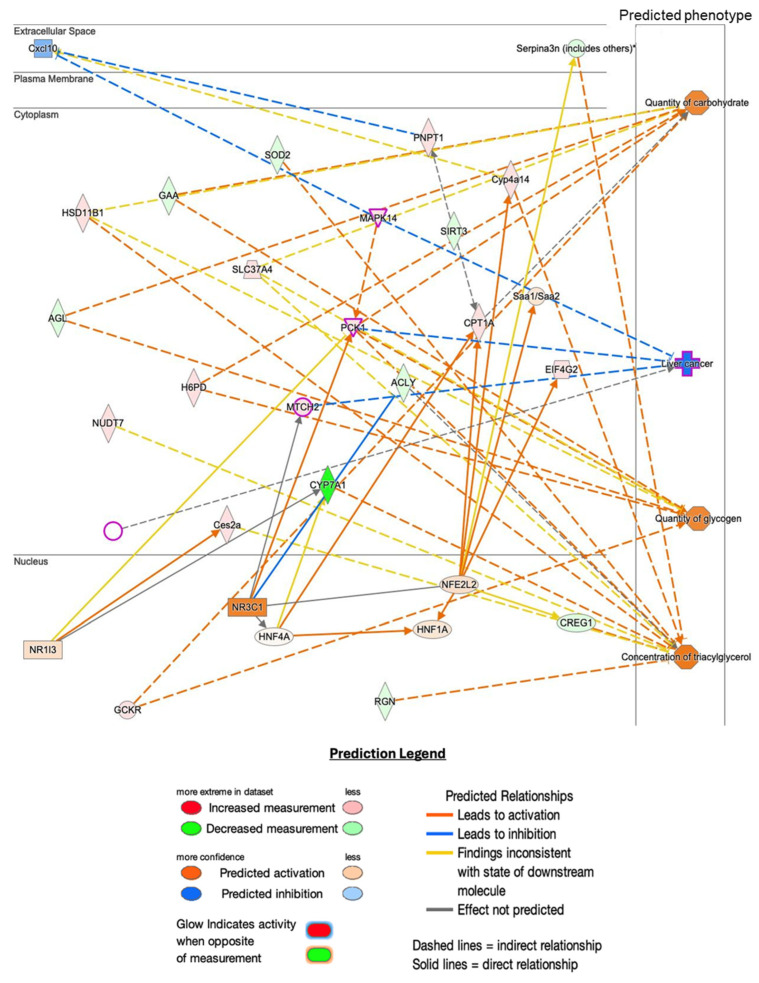
Pathway analysis of proteomics data. Predicted downstream physiological changes from differentially expressed proteins were analyzed using Qiagen IPA software 24.0.2. The panel presents predicted diseases/changes (z-score > 1.5) and the top proteins in the dataset affecting them (z-score > 1.99). * indicates other members of the Serpina family.

Ingenuity Pathway Analysis was used to map differentially expressed proteins (Figure 6). Similarly to RNA analysis, proteomic network analysis highlighted significant effects on liver health and metabolism, with shifts in cellular phenotypes and processes related to lipid metabolism, bile acid synthesis, oxidative stress response, and inflammatory pathways.

This analysis predicted several phenotypic changes associated with liver function after Hnf1aos1 knockdown. A significant decrease in bile acid synthesis was anticipated due to the downregulation of CYP7A1, a key enzyme in this pathway. Lipid metabolism was also notably altered, with evidence indicating decreased de novo lipogenesis and increased cholesterol efflux, reflecting disruptions in hepatic lipid regulation. Additionally, an enhanced oxidative stress response was predicted, driven by the upregulation of detoxification enzymes, such as GSTP1, which may serve as a compensatory mechanism to mitigate cellular stress. Furthermore, inflammatory responses appear to be modulated by changes in proteins, such as HSD11B1, suggesting potential shifts in glucocorticoid metabolism and inflammatory signaling pathways.

Collectively, these findings underscore the broad impact of Hnf1aos1 knockdown on liver metabolic homeostasis and cellular function. Collectively, these results demonstrated that Hnf1aos1 knockdown induces widespread changes in protein expression across key metabolic and regulatory pathways in the liver, further underscoring its critical role in maintaining hepatic homeostasis.

### 2.4. Shared Networks Between mRNA and Protein Expression Analysis

**Figure 7:**
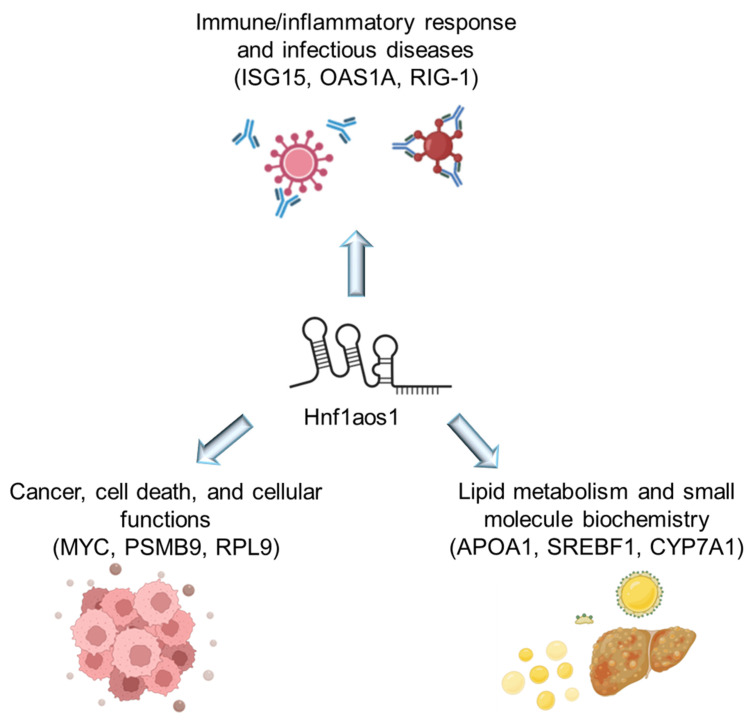
Shared networks between RNA-seq and proteomics. The shared networks identified through RNA-seq and proteomics analyses are illustrated.

A comprehensive overview of the shared networks between RNA-seq and proteomics analyses is presented in (Figure 7), highlighting three key areas of cellular function affected by the experimental conditions. In the lipid metabolism and small molecule biochemistry network, 32 mRNAs and 19 proteins were found to be differentially expressed, including APOA1, SREBF1, and LPL. The immune/inflammatory response and infectious disease network showed 32 mRNAs and 17 proteins with altered expression, with key proteins such as ISG15, OAS1A, and RIG-1. In the cancer, cell death, and cellular function network, 19 mRNAs and 19 proteins were differentially expressed, including MYC, PSMB9, and RPL9. All three networks met the significance threshold (Score = −log_10_(*p*-value). These results indicate that Hnf1aos1 knockdown affects multiple interconnected biological processes at both the transcriptomic and proteomic levels.

### 2.5. Histopathological Analysis and Predicted Metabolic Impacts on Liver Health

**Figure 8:**
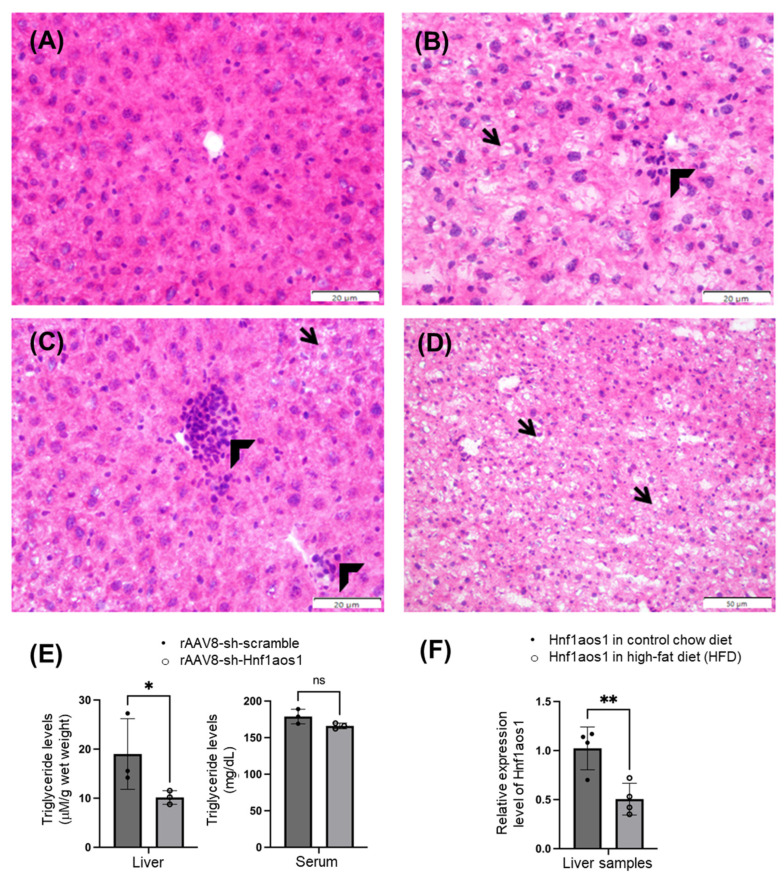
Hnf1aos1 knockdown induces hepatic pathological changes and alters lipid metabolism. (**A**) Normal liver from scramble mice, exhibiting intact hepatic architecture and well-organized cellular structures (H&E, 40×, bar = 20 μm). (**B**–**D**) Liver sections from Hnf1aos1 knockdown mice demonstrate significant pathological alterations, including disruption of hepatic cord architecture (**B**,**D**), indicative of compromised liver functions and structural integrity. Notably, hepatocellular vacuolar degeneration is observed (arrow), suggesting intracellular accumulation of lipids or other metabolites, which is characteristic of hepatocellular stress or injury. Inflammatory infiltration by mononuclear and granulocytic cells is prominent (arrowhead, (**B**,**C**)), indicating an ongoing inflammatory response within the liver. ((**B**,**C**): H&E, 40×, bar = 20 μm; (**D**): H&E, 20×, bar = 50 μm). (**E**) This bar graph displays the triglyceride levels measured via absorbance units (AUs) in both the liver and serum in the two groups: rAAV8-sh-scramble and rAAV8-sh-Hnf1aos1. (**F**) This bar graph shows the relative RNA expression of Hnf1aos1 in control chow diet and high-fat diet (HFD) conditions. Data are presented as mean ± SD. Statistical significance was determined by two-tailed unpaired *t*-test (**E**) and one-tailed unpaired *t*-test (**F**); * *p* < 0.05 and ** *p* < 0.01.

Hematoxylin and eosin (H&E) staining of the liver sections demonstrated profound alterations in the hepatic architecture. Normal liver sections from scrambled control mice (Figure 8A) showed well-organized hepatic cords and cellular structures. However, liver sections from Hnf1aos1 knockdown mouse revealed the significant disorganization of the hepatic cords (Figure 8B,D), along with hepatocellular vacuolar degeneration (Figure 8B, arrow), which may suggest intracellular lipid or metabolite accumulation, potentially indicating hepatic stress or injury. Moreover, inflammatory infiltration by mononuclear and granulocytic cells was observed (arrowheads in Figure 8B,C), indicating an ongoing inflammatory response within the liver, which may indicate an attempt to counteract the liver damage caused by metabolic or structural disruptions. These histopathological changes highlight the detrimental effects of Hnf1aos1 knockdown on liver function and structural integrity.

The differential expression of the Hnf1aos1 lncRNA was observed in mouse liver samples fed with a low-fat diet (LFD, *n* = 4) versus a high-fat diet (HFD, *n* = 4) (Figure 8E). The results indicated that Hnf1aos1 expression was significantly lower in the HFD group than in the LFD group (*p* < 0.01). The relative mRNA expression in the LFD group was approximately 1.0, whereas in the HFD group, it was approximately 0.5.

The triglyceride levels in the liver were measured using an absorbance-based assay (Figure 8F). The results revealed a significant decrease (*p* < 0.05) in triglyceride levels in the rAAV8-Hnf1aos1 group compared to the rAAV8-scramble control group. The rAAV8-scramble group showed triglyceride levels at around 20 Absorbance Units (AUs), while the rAAV8-Hnf1aos1 group exhibited levels at around 10 AUs. This reduction in triglyceride accumulation following Hnf1aos1 knockdown suggests the complex relationship between this lncRNA and triglyceride levels in the liver.

Overall, these findings underscore the critical role of Hnf1aos1 in maintaining liver metabolic homeostasis and modulating immune responses. The knockdown of this lncRNA leads to significant transcriptional and proteomic changes that affect key metabolic and inflammatory pathways.

## 3. Discussion

Our study demonstrates that Hnf1aos1 plays a critical role in regulating liver function and maintaining hepatic health. By knocking down Hnf1aos1 in both in vitro and in vivo models, we revealed its significant impact on metabolic and immune-related pathways in the liver. This aligns with emerging evidence that antisense lncRNAs, such as HNF1A-AS1 in humans, act as master regulators of metabolic and inflammatory liver diseases by modulating transcription factor stability and chromatin interactions [19].

### 3.1. Hnf1aos1 Knockdown Disrupts Hepatic Homeostasis

Our findings indicate that Hnf1aos1 knockdown significantly disrupted hepatic homeostasis. The 40% reduction in Hnf1aos1 RNA levels achieved by rAAV8-shRNA in vivo (Figure 1F) led to widespread transcriptional and proteomic changes that affected key metabolic genes and pathways involved in bile acid synthesis, lipid metabolism, and drug processing. These results mirror recent work illustrating that HNF1A-AS1 knockdown in human hepatocytes similarly disrupts HNF1A-driven metabolic networks, suggesting conservative regulatory mechanisms across species [9,19,21,25]. Differential expression analysis revealed notable changes in key hepatic regulators (Figure 4A) and their functions. The significant downregulation of SREBF1, SCD1, FASN, and VLDLR suggests a suppression of de novo lipogenesis and lipid uptake [34,35,36], while the upregulation of ABCA1 and APOB suggests the enhanced export of cholesterol from hepatocytes to lipid-poor apolipoproteins, and changes in the assembly and secretion of lipoproteins [37]. The downregulation of CAR and PXR indicates a potential reduction in the capacity of the liver to sense and respond to xenobiotics and drugs [38,39]. The upregulation of CYP7B1 suggests alterations in bile acid synthesis pathways [40,41]. The significant upregulation of LPL, APO19A, and APO19B indicates changes in lipoprotein processing and remodeling [42]. The increased expression of SOCS3 and STAT3, coupled with FOXP1 upregulation, suggests an activated inflammatory and immune response within the liver [43,44,45]. Opposing changes in VPS13D (downregulation) and VMP1 (upregulation) suggest altered vesicle trafficking and autophagy [46,47]. The downregulation of ACLY indicates a potential reduction in cytosolic acetyl-CoA production, which affects lipid and glucose metabolism [48,49]. The upregulation of GSTP1 suggests an increase in the detoxification of electrophilic compounds and protection against oxidative stress [50]. The log_2_ fold changes reflect the extent of upregulation or downregulation, highlighting a complex network of genes influencing hepatic metabolism. Furthermore, RT-qPCR analysis validates the changes in mRNA expression observed in the RNA-seq analysis (Figure 4B), confirming the significant downregulation of *Fasn*, *Vps13d*, *Vldlr*, and *Srebf1*, and significant upregulation of *Lpl*, *Shp-1*, *Apob*, *Abca1*, and *Vmp1* expression after Hnf1aos1 knockdown, which highlights the impact of Hnf1aos1 on lipid metabolism and the related pathways.

### 3.2. Multi-Omics Analysis Reveals Key Regulatory Changes

Integrating RNA-seq, proteomic analysis, and histopathological examination provided novel insights into the Hnf1aos1 regulatory network. RNA-seq data revealed the significant downregulation of key lipogenic regulators, such as *Srebf1*, *Scd1*, *Fasn*, and *Vldlr*, indicating the suppression of de novo lipogenesis and lipid uptake (Figure 4A,B). Conversely, genes involved in cholesterol efflux (*Abca1* and *Apob*) were upregulated. This paradoxical outcome aligns with clinical and experimental studies showing that advanced MASLD progression is driven not by triglyceride accumulation, but by lipotoxicity from alternative lipid mediators (diacylglycerols [DAGs], ceramides) and impaired lipid-handling mechanisms, including reduced VLDLR expression, which limits lipid uptake but exacerbates lipotoxic stress and hepatocellular injury [51,52,53,54]. In line with these mechanistic shifts, we observed a significant reduction in hepatic triglyceride content in the Hnf1aos1-knockdown group. These changes were consistent with the observed decrease in liver triglyceride levels following Hnf1aos1 knockdown (Figure 8E), suggesting a complex relationship between this lncRNA and lipid metabolism. This parallels findings in MASLD patients, where HNF1A-AS1 expression inversely correlates with hepatic steatosis severity [55]. Proteomic analysis further corroborated these findings, revealing the significant downregulation of CYP7A1 and upregulation of APO19A (Figure 5D), indicating disrupted bile acid synthesis and lipid transport. These changes reflect the liver’s attempts to manage excess lipids and protect itself against lipotoxicity. The increased expression of OAS1A, OASL1, and OASL2 suggests that liver cells may be primed for a stronger antiviral response. In summary, Hnf1aos1 knockdown, especially in the context of HFD, may disrupt the interconnected pathways of lipid accumulation, inflammation, and metabolic dysregulation, affecting liver function and overall metabolic health. Regarding CYP7A1, the observed fold change in protein abundance between groups was 0.00000003 (3 × 10^−8^), indicating an extremely strong reduction. While this dramatic decrease was statistically significant by our analysis pipeline, we acknowledge that such a large fold change may reflect a combination of true biological suppression and technical limitations, such as quantification floor effects in proteomics. Therefore, we caution that although the reduction in CYP7A1 protein is robust and consistent with the expected direction of effect, the precise magnitude of change should be interpreted with care. Further validation using orthogonal methods, such as targeted proteomics or immunoblotting, will be necessary to confirm the extent of CYP7A1 depletion observed in this study. To ensure robustness, all proteomics data were filtered for a 1% false discovery rate (FDR) at both peptide and protein levels, and only proteins quantified in at least two biological replicates per group were included. Differential abundance was assessed using the MaxQuant/Perseus pipeline with Benjamini–Hochberg correction (FDR < 0.05).

### 3.3. Implications for Liver Diseases: Shared Pathways and Impact on Metabolic Dysfunction-Associated Steototic Liver Disease (MASLD)

Our study employed a comprehensive multi-omics approach, integrating RNA-seq and proteomic data to identify shared pathways that are significantly affected by Hnf1aos1 knockdown. This analysis, summarized in Figure 7, revealed the complex relationship of biological processes influenced by this lncRNA and its implications in liver diseases, particularly MASLD, and its progression to metabolic dysfunction-associated steatohepatitis (MASH) and hepatocellular carcinoma (HCC).

One of the most striking observations was the downregulation of triglyceride levels in the liver upon Hnf1aos1 knockdown (Figure 8E). We also observed downregulated Hnf1aos1 expression in high-fat diet (HFD) conditions compared to control chow diet conditions (Figure 8F), suggesting that Hnf1aos1 may be a critical mediator in the liver’s response to increased dietary fat intake. However, we also observed compensatory responses that appeared to make the liver more efficient in lipid management.

In addition to the observed reduction in hepatic triglyceride levels, our transcriptomic and proteomic analyses revealed several compensatory responses that may enhance hepatic lipid management following Hnf1aos1 knockdown. Notably, genes involved in cholesterol efflux and lipoprotein assembly, such as ABCA1 and APOB, were upregulated, suggesting an adaptive increase in the export of cholesterol and triglycerides from hepatocytes into the bloodstream. The upregulation of LPL (lipoprotein lipase) and APOE further supports enhanced peripheral lipid clearance and the remodeling of circulating lipoproteins. These compensatory changes are consistent with previous reports demonstrating that the activation of reverse cholesterol transport and lipoprotein remodeling pathways can mitigate hepatic steatosis and protect against lipotoxicity [56,57].

Collectively, these findings suggest that the loss of Hnf1aos1 triggers a coordinated hepatic response to maintain lipid homeostasis, characterized by increased lipid export, enhanced lipoprotein remodeling, and improved peripheral lipid clearance. This adaptive response may initially protect against triglyceride accumulation, but could also predispose to altered lipid profiles and increased circulating lipids, as observed in advanced MASLD and NAFLD patients [56,58]. Thus, our data highlight the dual nature of these compensatory mechanisms, offering short-term protection against steatosis, while potentially contributing to systemic dyslipidemia and the progression of metabolic liver disease.

### 3.4. The Shared Pathways Analysis Revealed Several Key Areas Affected by Hnf1aos1 Knockdown

Lipid Metabolism and Small Molecule Biochemistry: We observed the extensive dysregulation of genes and proteins involved in lipid transport, cholesterol metabolism, fatty acid synthesis, and bile acid pathways. Key dysregulated genes included APOA1, SREBF1, and CYP7A1, along with additional players such as APOA2, LPL, FASN, ABCA1, VLDLR, CYP7B1, CYP4A10, CYP4A12A, and members of the ACOT families. The combined impact of these dysregulations suggests that reduced Hnf1aos1 expression under HFD conditions may impair the liver’s ability to manage triglycerides properly, potentially contributing to MASLD development or progression. Following Hnf1aos1 knockdown, the extensive dysregulation of genes involved in lipid transport, fatty acid synthesis, and cholesterol metabolism would likely exacerbate hepatic lipid accumulation, oxidative stress, and inflammation, accelerating MASLD progression. The impaired regulation of key pathways, such as reduced SREBF1-driven lipogenesis and altered cholesterol/bile acid homeostasis, could promote hepatocellular injury and fibrogenesis, increasing the risk of progression from simple steatosis to steatohepatitis and advanced liver disease. The downregulation of SREBF1 and its target lipogenic genes leads to decreased de novo lipogenesis, whereas upregulated cholesterol efflux mechanisms may serve as compensatory responses. Additionally, drug metabolism pathways showed the dysregulation of cytochrome P450 enzymes (CYP1A2, CYP2C70, AND CYP4A14), UDP-glucuronosyltransferases (UGT2B38 AND UGT2B1), and glutathione S-transferases (GSTM1 AND GSTM3), indicating a compromised detoxification capacity.

Immune/Inflammatory Response and Infectious Disease: We identified the significant dysregulation of genes and proteins involved in interferon signaling, antiviral response, and complement activation. The network prominently featured ISG15, OAS1A, and Rigi (RIG-I), along with additional antiviral mediators including OAS2, OAS3, OASL2, IRF2BP2, C4B, C4BP, PARP14, and IFIH1 (MDA5). Recent studies have suggested that viral infections can exacerbate MASLD, and that the activation of these mechanisms may be a double-edged sword, where chronic inflammation could worsen liver injury and metabolic dysregulation. The upregulation of these immune response pathways suggests that Hnf1aos1 knockdown may prime the liver for enhanced inflammatory responses.

Cancer, Cell Death, and Cellular Function: This network highlighted the dysregulation of genes critical for cell proliferation, apoptosis, and protein homeostasis. Key players included MYC, PSMB9, and RPL9, representing oncogenic signaling, proteasome function, and ribosomal protein synthesis, respectively. Additional dysregulated components involved protein quality control mechanisms, including ER stress response proteins (PDIA3, PDIA4, and HSPA5), ubiquitin–proteasome system components (PSMD8, PSMD14, and RNF213), and translation machinery (RPS9, EEF1A2, and EIF4A1). The impaired ability of cells to manage misfolded proteins and maintain proper protein synthesis due to Hnf1aos1 knockdown may contribute to cellular stress and altered cell fate decisions, potentially influencing both hepatocyte survival and proliferation.

Notably, while murine-specific enzymes lack direct human orthologs, the regulatory relationship between Hnf1aos1 and HNF1A, a conserved transcriptional regulator, suggests translational relevance. Human orthologs fulfill analogous roles, underscoring the potential for conserved pathway regulation despite species differences in enzyme isoforms. The comprehensive view provided by this three-network analysis underscores the complex role of Hnf1aos1 in regulating liver function and its potential involvement in various liver-related diseases. Considering these interconnected pathways, monitoring Hnf1aos1 levels could provide valuable insights into disease states and progression, positioning it as a potential therapeutic target and biomarker for liver disease.

### 3.5. Future Directions

Future studies should validate these findings in humanized models or primary human hepatocytes to address species-specific differences in drug metabolism and confirm translational relevance. Further studies should also focus on investigating the precise molecular mechanisms through which Hnf1aos1 regulates bile acid synthesis and lipid metabolism. Elucidating the direct transcriptional targets and influencing pathways is critical for understanding its regulatory networks. Further research on Hnf1aos1’s role in hepatic lipid homeostasis and immune modulation could enhance the potential of personalized medical approaches. This could potentially lead to tailored therapeutic strategies based on individual liver metabolic profiles to improve the management of liver diseases and metabolic disorders.

## 4. Materials and Methods

### 4.1. Cell Culture, Treatment, and Transient Transfection

Hepa-1c1c7, a mouse hepatoma cell line, was obtained from American Type Culture Collection (ATCC, Manassas, VA, USA). Cells were cultured in alpha-modified Eagle’s medium (AMEM; Thermo Fisher Scientific, Waltham, MA, USA) supplemented with 10% fetal bovine serum (FBS; Gibco, Thermo Fisher Scientific) at 37 °C in a humidified atmosphere with 5% CO_2_. For transfection experiments, cells were seeded 24 h prior to treatment at a density that would achieve 50–60% confluence at the time of transfection. Cells were transfected with either scrambled siRNA (Catalog #4390843) or Hnf1aos1-specific siRNA (5′-TACTGTAAAGCGTGTTTATTAAC-3′) (Dharmacon, Lafayette, CO, USA) at a final concentration of 50 nM using Lipofectamine RNAiMAX reagent (Invitrogen, Thermo Fisher Scientific) according to the manufacturer’s instructions. The Hnf1aos1-specific siRNA was designed using the siDIRECT software v2.1 (http://sidirect2.rnai.jp/, accessed on 25 September 2023).

### 4.2. Viral Plasmids and Virus Production for Animal Treatment

Recombinant AAVs (rAAVs) for both the control (rAAV8-Scramble) (5′-CCTAAGGTTAAGTCGCCCTCGCTCGAGCGAGGGCGACTTAACCTTAGG-3′) and knockdown (rAAV8-Hnf1aos1) (5′-TACTGTAAAGCGTGTTTATTACTCGAGTAATAAACACGCTTTACAGTA-3′) groups were purchased from VectorBuilder (Chicago, IL). The same siRNA target for Hnf1aos1 was used for shRNA. C57BL/6J mice were obtained from The Jackson Laboratory (Bar Harbor, ME, USA) and maintained under standard conditions (12 h light/dark cycle, 22 ± 2 °C, 50–60% humidity) with ad libitum access to food and water. Twenty male mice, approximately six weeks of age, were randomly divided into two groups (*n* = 10 per group). Mice were injected with 100 μL of either AAV-Hnf1aos1 or AAV-scramble (1 × 1012 vector genomes/mL) through the tail vein. The AAV-Hnf1aos1 construct contained a shRNA targeting the same sequence as that of the siRNA used in the in vitro experiments. Four weeks post injection, the mice were weighed and euthanized via CO_2_ inhalation, followed by cervical dislocation. Blood was collected via cardiac puncture, and liver and kidney samples were harvested. Liver tissue samples were flash-frozen in liquid nitrogen for RNA and protein analysis, whereas the others were embedded in Optimal Cutting Temperature (OCT) compound for histological examination. All animal procedures were approved by the Institutional Animal Care and Use Committee (IACUC) at the University of Connecticut and conducted in accordance with the NIH Guide for the Care and Use of Laboratory Animals.

### 4.3. Quantification of Gene Expression in In Vitro Cells and In Vivo Mice via RT-qPCR

The total RNA was isolated from cultured cells and liver tissue using TRIzol reagent (Thermo Fisher Scientific) according to the manufacturer’s protocol. Liver samples were homogenized in TRIzol using a Precellys 24 tissue homogenizer (Bertin Technologies, Montigny-le-Bretonneux, France). The RNA concentration and purity were assessed using a NanoDrop spectrophotometer (Thermo Fisher Scientific). Complementary DNA (cDNA) was synthesized from 1 μg of total RNA using the iScript cDNA Synthesis Kit (Bio-Rad, Hercules, CA, USA). Quantitative PCR was performed on a CFX96 Real-Time System (Bio-Rad, Hercules, CA, USA) using iTaq Universal SYBR Green Supermix (Bio-Rad) and gene-specific primers (Integrated DNA Technologies, Coralville, IA, USA). The primer sequences used are listed in Table 1. Gene expression was normalized to glyceraldehyde-3-phosphate dehydrogenase (GAPDH) as an internal control. Relative expression levels were calculated using the 2^−ΔΔCt^ method and expressed as the fold change relative to the control samples. All primers used in this study were purchased from IDT (Fall River, MA, USA) (Table 1).

### 4.4. Transcriptomic Analysis via RNA-Seq

The total RNA was extracted from liver samples of six mice (three scramble-treated and three Hnf1aos1-treated mice). RNA samples were processed using Genewiz (South Plainfield, NJ, USA), including DNase treatment to remove genomic DNA contamination. RNA quality and quantity were assessed using a NanoDrop spectrophotometer (Thermo Fisher Scientific) and Agilent Bioanalyzer 2100 (Agilent Technologies, Santa Clara, CA, USA). Samples with RNA Integrity Numbers (RINs) greater than eight were selected for sequencing. RNA sequencing libraries were prepared using an Illumina TruSeq Stranded mRNA Library Prep Kit. Briefly, the mRNA was enriched by poly(A) selection, fragmented, and primed for cDNA synthesis. Double-stranded cDNA was synthesized, end-repaired, and ligated using Illumina sequencing adapters. Libraries were PCR-amplified and sequenced on an Illumina NovaSeq 6000 platform, generating 150 bp paired-end reads. Raw sequencing reads were assessed for quality using FastQC (v0.11.9). Adapter sequences and low-quality bases were trimmed using Trimmomatic (v0.39). Clean reads were aligned to the mouse reference genome (GRCm38) using STAR (v2.7.3a). Gene-level reading counts were obtained using feature counts from the subread package (v2.0.1), considering only the uniquely mapped exonic reads. Differential gene expression analysis, which utilizes normalized counts as the input, was performed using DESeq2 (v1.30.0) in the R software (v4.0.3). Raw read counts for each gene were obtained using feature counts, and normalization was carried out via DESeq2 to account for differences in sequencing depth and RNA composition between samples. Genes with an adjusted *p*-value < 0.05 and |log_2_ fold change| > 1 were considered significantly differentially expressed. The functional annotation of differentially expressed genes was conducted using DAVID (v6.8) and KEGG pathway analyses. Pathway analysis was conducted using the Qiagen Ingenuity Pathway Analysis (IPA) software 24.0.2.

### 4.5. Untargeted Protein Identification and Label-Free Quantification via Tandem Mass Spectrometry

Protein concentrations in the liver tissue homogenates were quantified using a BCA assay. Aliquots (100 μg) were processed using S-Trap micro columns (ProtiFi, LLC) with modifications to the manufacturer’s protocol. Briefly, the samples were reduced with DTT (25 °C, 1 h), alkylated with iodoacetamide (25 °C, 30 min, dark), acidified with phosphoric acid, and diluted 6-fold with binding/wash buffer (90% methanol, 100 mM TEAB). After loading and washing, the proteins were digested on a column with trypsin/LysC (1:10 *w*/*w*, Promega #V5073) overnight at 25 °C. The peptides were eluted, desalted using Pierce peptide desalting spin columns (Thermo Fisher #89852), and resuspended in 0.1% formic acid in water (Solvent A). Samples were normalized to 1 μg of the total peptides for injection based on A280 absorbance. The peptides were analyzed using a Thermo Scientific Ultimate 3000 RSLCnano UPLC coupled with a Thermo Scientific Eclipse Tribrid Orbitrap mass spectrometer. Separation was performed on a nanoEase M/Z Peptide BEH C18 column (1.7 μm, 75 μm × 250 mm, Waters) using a 120 min gradient of 4–90% Solvent B (0.1% formic acid in acetonitrile) at 300 nL/min. The mass spectrometer was operated in positive mode with nanoflow electrospray ionization (capillary voltage, 2200 V). MS1 scans were acquired at a resolution of 120,000 (AGC target 4e5, maximum ion time Auto, RF lens 30%, scan range 300–1800 m/z). Data-dependent MS2 scans were acquired in the Orbitrap (resolution 15,000, intensity threshold 5.0e4, AGC target standard, maximum ion time auto, isolation window 1.6 m/z, and 3 s cycle time). HCD fragmentation was performed at an energy of 30%. Dynamic exclusion was set to 30 s after one observation for charge states 2–8. Peptide identification and label-free quantification were performed using MaxQuant (v2.0.2.0) and Andromeda search engines. Raw data were searched against the UniProt Mus Musculus reference proteome (UP000000589, accessed on 1 October 2024) and MaxQuant contaminants database. Search parameters included a minimum peptide length of 5 residues, maximum mass of 4600 Da, up to five variable modifications (Met oxidation, protein N-terminal acetylation, Asn/Gln deamidation, N-terminal Gln to pyroGlu), fixed Cys carbamidomethylation, and trypsin/P specificity, with up to two missed cleavages. The results were filtered to obtain a 1% FDR at the peptide and protein levels. The MaxQuant output was imported into Scaffold (version 5.3.4, manufactured by Proteome Software, Inc., Portland, OR, USA) for visualization and analysis.

### 4.6. H&E Staining

Liver samples embedded in an Optimal Cutting Temperature (OCT) medium were sectioned at a 6 μm thickness using a cryostat (Leica CM3050 S). The sections were mounted on Super frost Plus microscope slides and allowed to air dry for 30 min at room temperature. H&E staining was performed by immersing the slides in filtered Harris Hematoxylin (Lerner Laboratories Pittsburgh, PA, USA) for 10 s, followed by rinsing with running tap water until they were clear. The sections were then immersed in a 1% aqueous Eosin Y solution (prepared with Sigma #E-6003) for 30 s and rinsed again in running tap water until they were clear. The sections were dehydrated by passing through a graded ethanol series (50%, 70%, 80%, 95% × 2, and 100% × 2) using reagent-grade alcohol, and then cleared in xylene (Fisher #HC700-1GAL) with 3–4 changes. Finally, coverslips were mounted using Permount (Fisher SP15-100) as the mounting medium. Stained sections were examined under a light microscope; nuclei and other basophilic structures appeared blue, whereas cytoplasm and acidophilic structures were stained light to dark red.

### 4.7. Quantification of TG

The TG levels were measured using a Triglyceride Colorimetric Assay Kit (Thermo Fisher Scientific, EEA0281). The assay was performed according to the manufacturer’s instructions. Briefly, samples from each group and standards were prepared in microplate wells. The TG reaction mix was added to each well and incubated at room temperature for 30 min. Absorbance was measured at 570 nm using a microplate reader. TG concentrations were calculated by comparing the sample absorbance values to a standard curve generated with known TG concentrations. All samples were assayed in triplicate, and the average values were used for analysis.

### 4.8. Statistical Analysis

Data are presented as mean ± standard deviation (SD). Statistical analyses were performed using GraphPad Prism version 9.0 (GraphPad Software, San Diego, CA, USA). An unpaired two-tailed *t*-test was used to compare the two groups. Statistical significance is defined as follows: * *p* < 0.05, ** *p* < 0.01, and *** *p* < 0.001. In RNA-seq data analysis, differential gene expression was determined using DESeq2, with genes considered significantly differentially expressed at an adjusted *p* < 0.05 and an absolute log_2_ fold change > 1. For proteomic data, the MaxQuant output was filtered to 1% FDR at the peptide and protein levels, and pathway analyses were conducted using Qiagen Ingenuity Pathway Analysis (IPA) software, with statistical significance determined using Benjamini–Hochberg-corrected *p*-values.

## Figures and Tables

**Table 1:** Primers used for reverse transcription and PCR in the study.

Gene	Forward Primer (5′→3′)	Reverse Primer (5′→3′)
*Hnf1aos1*	GACTGTCCCCAAACTCCTCA	TTCCCTGAGATTATCGCCCG
*Fasn*	CACAGTGCTCAAAGGACATGCC	CACCAGGTGTAGTGCCTTCCTC
*Lpl*	GCGTAGCAGGAAGTCTGACCAA	AGCGTCATCAGGAGAAAGGCG
*Shp-1*	TGGTTTCACCGGGACCTCAGC	AGTAAGGCTGCCGCAGGTAGA
*Apol9a*	GTGGATAGGATTGCCAGCAAG	AGAGGGGTTCCTTTCAGACTG
*Apol9b*	GTAGCTAGGATTGTCAACAAGA	CAGAGGGGTTCCTTCCAGCC
*Abca1*	GGAGCCTTTGTGGAACTCTTCC	CGCTCTCTTCAGCCACTTTGAG
*Vmp1*	GTTGGTCTTGGAACAGGACTGC	ATCTGGTCAGGATAGGGTGGCT
*Vps13d*	AAAGTGACCCTCCAGATTCCT	CGCTTTCTTACGCTCCCTCT
*Vldlr*	ACGGCAGCGATGAGGTCAACTG	CAGAGCCATCAACACAGTCTCG
*Srebf1*	CGACTACATCCGCTTCTTGCAG	CCTCCATAGACACATCTGTGCC

## Data Availability

All raw data and processed data are stored on the OneDrive of Zhong laboratory at the University of Connecticut. The data are available to the public. The individual repository accession numbers are listed below. When published, all raw data and processed data were deposited to a NIGMS dedicated repository followed by the NIGMS Data Management and Sharing Plan policy. (1). RNA-seq data have been uploaded to NCBI GEO, GSE284652. (2). The Proteomics Data has been uploaded to the ProteomeXchange Consortium Via the PRIDE partner repository, PXD063297. The data can be accessed by logging into PRIDE with the following account details: Username: reviewer_pxd063297@ebi.ac.uk, and Password: GwEyeiF3eDJK. (3). RT-qPCR data have been uploaded to Mendeley Data, DOI: 10.17632/svs5p2s3wj.1. (4). Histology files have been uploaded to Mendeley Data, DOI: 10.17632/cr3hj2w6th.1. (5). Absorbance files have been uploaded to Mendeley Data, DOI: 10.17632/rxw5sv52dw.1.

## Supplementary Materials

The following supporting information can be downloaded at: https://www.mdpi.com/article/10.3390/ncrna11040052/s1. Figure S1: Differential gene expression analysis of OAS genes. This bar plot illustrates the log_2_ fold change in expression levels of eight OAS genes. Each bar represents the magnitude of gene expression changes, with the log_2_ fold change values displayed inside the bars. The significance of differential expression is indicated by stars positioned outside the bars: *** *p* < 0.001, ** *p* < 0.01, and * *p* < 0.05.

## Abbreviations

Abca1, ATP-binding cassette transporter A1; Abcd2 (ALDR), Adrenoleukodystrophy protein; Acly, ATP Citrate Lyase; ACOT1, Acyl-CoA Thioesterase 1; Agpat, 1-Acyl-sn-Glycerol-3-Phosphate Acyltransferase; ALDR (Abcd2), Adrenoleukodystrophy protein; AMEM, Alpha Modification of Eagle’s Medium; Apoa1, Apolipoprotein A1; Apoa2, Apolipoprotein A2; APOB, Apolipoprotein B; Apol9a, Apolipoprotein L9a; Apol9b, Apolipoprotein L9b; APOOL, Apolipoprotein L; ATCC, American Type Culture Collection; C4b, Complement component 4b; C4bp, Complement component 4b binding protein; CAR (NR1I3), Constitutive Androstane Receptor (Nuclear Receptor Subfamily 1 Group I Member 3); CAG, Cytomegalovirus early enhancer/chicken β-actin (CBA) promoter with rabbit β-globin splice acceptor; cDNA, Complementary DNA; Ces2a, Carboxylesterase 2A; Ces2b, Carboxylesterase 2B; Ces4a, Carboxylesterase 4A; CTSB, Cathepsin B; CTSD, Cathepsin D; CTSL, Cathepsin L; CTSS, Cathepsin S; Cyp21a1, Cytochrome P450 Family 21 Subfamily A Member 1; Cyp2a12, Cytochrome P450 Family 2 Subfamily A Member 12; Cyp2c70, Cytochrome P450 Family 2 Subfamily C Member 70; Cyp4a10, Cytochrome P450 Family 4 Subfamily A Member 10; Cyp4a12a, Cytochrome P450 Family 4 Subfamily A Member 12A; Cyp4a14, Cytochrome P450 Family 4 Subfamily A Member 14; Cyp4a31, Cytochrome P450 Family 4 Subfamily A Member 31; CYP7A1, Cytochrome P450 Family 7 Subfamily A Member 1; Cyp7a1, Cytochrome P450 Family 7 Subfamily A Member 1; CYP7B1, Cytochrome P450 Family 7 Subfamily B Member 1; Cyp7b1, Cytochrome P450 Family 7 Subfamily B Member 1; DANCR/ANCR, Differentiation-Antagonizing Non-Protein Coding RNA; DAVID, Database for Annotation, Visualization and Integrated Discovery; DILC, Downregulated in Liver Cancer; DNAJC, DnaJ Heat Shock Protein Family C Member; Dnaja1, DnaJ Heat Shock Protein Family A Member 1; Dnaja10, DnaJ Heat Shock Protein Family A Member 10; Dnajc12, DnaJ Heat Shock Protein Family C Member 12; Dnail, DnaJ Heat Shock Protein Family A Member 1; DTT, Dithiothreitol; EEF, Eukaryotic Elongation Factor; Eef1a2, Eukaryotic Elongation Factor 1 Alpha 2; Eef1b2, Eukaryotic Elongation Factor 1 Beta 2; Eef1g, Eukaryotic Elongation Factor 1 Gamma; Eef2, Eukaryotic Elongation Factor 2; EIF, Eukaryotic Initiation Factor; Eif4a1, Eukaryotic Initiation Factor 4A1; Eif4e3, Eukaryotic Initiation Factor 4E3; Eif4h, Eukaryotic Initiation Factor 4H; Eif5a, Eukaryotic Initiation Factor 5A; Eps812, EPS8 Like 2; ER, Endoplasmic Reticulum; EZH2, Enhancer of zeste homolog 2; Fasn, Fatty Acid Synthase; FBS, fetal bovine serum; FDR, False Discovery Rate; FENDRR, Functional Expression of Non-coding RNA in Development and Reproduction; Foxp1, Forkhead Box P1; G3BP2, Ras GTPase-Activating Protein Binding Protein 2; GAPDH, glyceraldehyde-3-phosphate dehydrogenase; GAS5, Growth Arrest Specific 5; GO, Gene Ontology; GPR146, G protein-coupled receptor 146; Gstm1, Glutathione S-Transferase Mu 1; Gstm3, Glutathione S-Transferase Mu 3; Gstm5, Glutathione S-Transferase Mu 5; Gstm6, Glutathione S-Transferase Mu 6; GSTP1, Glutathione S-Transferase Pi 1; H&E, Hematoxylin and eosin; H19, Imprinted Maternally Expressed Transcript; HAP1, Huntingtin Associated Protein 1; HCC, Hepatocellular Carcinoma; HCD, Higher-energy Collisional Dissociation; HEIH, High Expression In HCC; HFD, high-fat diet; HNF1A, Hepatocyte Nuclear Factor 1α; HNF1A-AS1, HNF1A antisense RNA 1; Hnf1aos1, Hnf1a opposite RNA 1; HOTAIR, HOX transcript antisense intergenic RNA; HOTTIP, HOXA transcript at the distal tip; HP, Haptoglobin; HPX, Hemopexin; HSPA5, Heat Shock 70kDa Protein 5; HULC, Highly Upregulated in Liver Cancer; IACUC, Institutional Animal Care and Use Committee; IFIH1 (MDA5), Interferon Induced With Helicase C Domain 1; IPA, Ingenuity Pathway Analysis; IRF2BP2, Interferon Regulatory Factor 2 Binding Protein 2; Isg15, Interferon-Stimulated Gene 15; IVIS, In Vivo Imaging System; KEGG, Kyoto Encyclopedia of Genes and Genomes; LMFL, Lipase Maturation Factor 1; lncRNA, Long non-coding RNA; Lpl, Lipoprotein Lipase; LYPLAL1, Lysophospholipase Like 1; MALAT1, Metastasis Associated Lung Adenocarcinoma Transcript 1; MEG3, Maternally Expressed Gene 3; MASLD, Metabolic dysfunction-Associated Steatotic Liver Disease; miRNAs, MicroRNAs; Mocos, Molybdenum Cofactor Sulfurase; MS, Mass Spectrometry; MALFD, Non-Alcoholic Fatty Liver Disease; MASH, Non-Alcoholic Steatohepatitis; NEAT1, Nuclear Enriched Abundant Transcript 1; NIH, National Institutes of Health; NR1I2 (PXR), Nuclear Receptor Subfamily 1 Group I Member 2 (Pregnane X Receptor); NR1I3 (CAR), Nuclear Receptor Subfamily 1 Group I Member 3 (Constitutive Androstane Receptor); NRPI, Neuropilin 1; Oas1a, 2′-5′-Oligoadenylate Synthetase 1A; Oas1c, 2′-5′-Oligoadenylate Synthetase 1C; Oas1g, 2′-5′-Oligoadenylate Synthetase 1G; Oas1l, 2′-5′-Oligoadenylate Synthetase 1L; Oas1b, 2′-5′-Oligoadenylate Synthetase 1B; Oas2, 2′-5′-Oligoadenylate Synthetase 2; Oas3, 2′-5′-Oligoadenylate Synthetase 3; Oasll, 2′-5′-Oligoadenylate Synthetase Like 1; Oasl2, 2′-5′-Oligoadenylate Synthetase Like 2; OCT, Optimal Cutting Temperature; OSBP, Oxysterol Binding Protein; PARP14, Poly(ADP-Ribose) Polymerase 14; Pdia3, Protein Disulfide Isomerase family A member 3; Pdia4, Protein Disulfide Isomerase family A member 4; Pdia6, Protein Disulfide Isomerase family A member 6; PRC2, Polycomb Repressive Complex 2; Prkaca, Protein Kinase cAMP-Activated Catalytic Alpha; PSMB/D, Proteasome Subunit Beta/Delta; Psmd14, Proteasome 26S Subunit Non-ATPase 14; Psmd2, Proteasome 26S Subunit Non-ATPase 2; Psmd8, Proteasome 26S Subunit Non-ATPase 8; PTPN6 (Shp-1), Protein Tyrosine Phosphatase Non-Receptor Type 6; PXR (NR1I2), Pregnane X Receptor (Nuclear Receptor Subfamily 1 Group I Member 2); qPCR, Quantitative PCR; rAAV, Recombinant Adeno-Associated Viruses; RF, Radio Frequency; RIGI (Rigi), Retinoic acid-inducible gene I; RIN, RNA Integrity Number; RNF, Ring Finger Protein; RNF125, Ring Finger Protein 125; RNF213, Ring Finger Protein 213; RP, Ribosomal Protein; Rp19, Ribosomal Protein S19; Rps9, Ribosomal Protein S9; Rpsa, Ribosomal Protein SA; RT-qPCR, Real-Time Quantitative PCR; Scd1, Stearoyl-CoA Desaturase 1; SF3B, Splicing Factor 3B; Sf3b2, Splicing Factor 3B Subunit 2; Sf3b3, Splicing Factor 3B Subunit 3; Sf3b4, Splicing Factor 3B Subunit 4; shRNA, short hairpin RNA; Shp-1 (PTPN6), SH2 domain-containing protein tyrosine phosphatase-1; siRNA, Small interfering RNA; Slc10a, Solute Carrier Family 10 Member A; Slc13a2, Solute Carrier Family 13 Member 2; Slc13a5, Solute Carrier Family 13 Member 5; Slc15a4, Solute Carrier Family 15 Member 4; Slc22a3, Solute Carrier Family 22 Member 3; Slc25a1, Solute Carrier Family 25 Member 1; Slc25a21, Solute Carrier Family 25 Member 21; Slc25a45, Solute Carrier Family 25 Member 45; Slc30a4, Solute Carrier Family 30 Member 4; Slc30a5, Solute Carrier Family 30 Member 5; Slc33al, Solute Carrier Family 33 Member 1; Slc36a4, Solute Carrier Family 36 Member 4; Slc37a4, Solute Carrier Family 37 Member 4; Slc39a14, Solute Carrier Family 39 Member 14; Slc39a4, Solute Carrier Family 39 Member 4; Slc39a7, Solute Carrier Family 39 Member 7; Socs3, Suppressor Of Cytokine Signaling 3; SREBF1, Sterol Regulatory Element-Binding Transcription Factor 1; Srprb, Signal Recognition Particle Receptor Subunit B; STAT3, Signal Transducer and Activator of Transcription 3; TEAB, Triethylammonium bicarbonate; TG, Triglyceride; TLR2, Toll-Like Receptor 2; TNFRSF1A, TNF Receptor Superfamily Member 1A; Trim25, Tripartite motif-containing protein 25; Ugt1a2, UDP Glucuronosyltransferase Family 1 Member A2; Ugt2b1, UDP Glucuronosyltransferase Family 2 Member B1; Ugt2b35, UDP Glucuronosyltransferase Family 2 Member B35; Ugt2b37, UDP Glucuronosyltransferase Family 2 Member B37; Ugt2b38, UDP Glucuronosyltransferase Family 2 Member B38; UPLC, Ultra Performance Liquid Chromatography; USOL, Unconventional SNARE in the ER Localization 1; Vldlr, Very Low-Density Lipoprotein Receptor; VMP1, Vacuole Membrane Protein 1; and VPS13D, Vacuolar Protein Sorting 13 Homolog D.
