# Supplementary material for: Unraveling the Regulatory Impact of LncRNA Hnf1aos1 on Hepatic Homeostasis in Mice
Source: Noncoding RNA. 2025 Jul 4;11(4):52. doi: 10.3390/ncrna11040052 (PMC12286052; doi:10.3390/ncrna11040052)
Supplement: Supplementary file 1 [file ncrna-11-00052-s001.zip › ncrna-3633611-supplementary.pdf]

Unraveling the Regulatory Impact of LncRNA HNF1AOS1 on Hepatic Homeostasis in Mice

Beshoy Armanios[1], Jing Jin[1], Holly Kolmel[1], Ankit P. Laddha[1], Neha Mishra[2], Jose E. Manautou[1], and Xiao-bo Zhong[1]

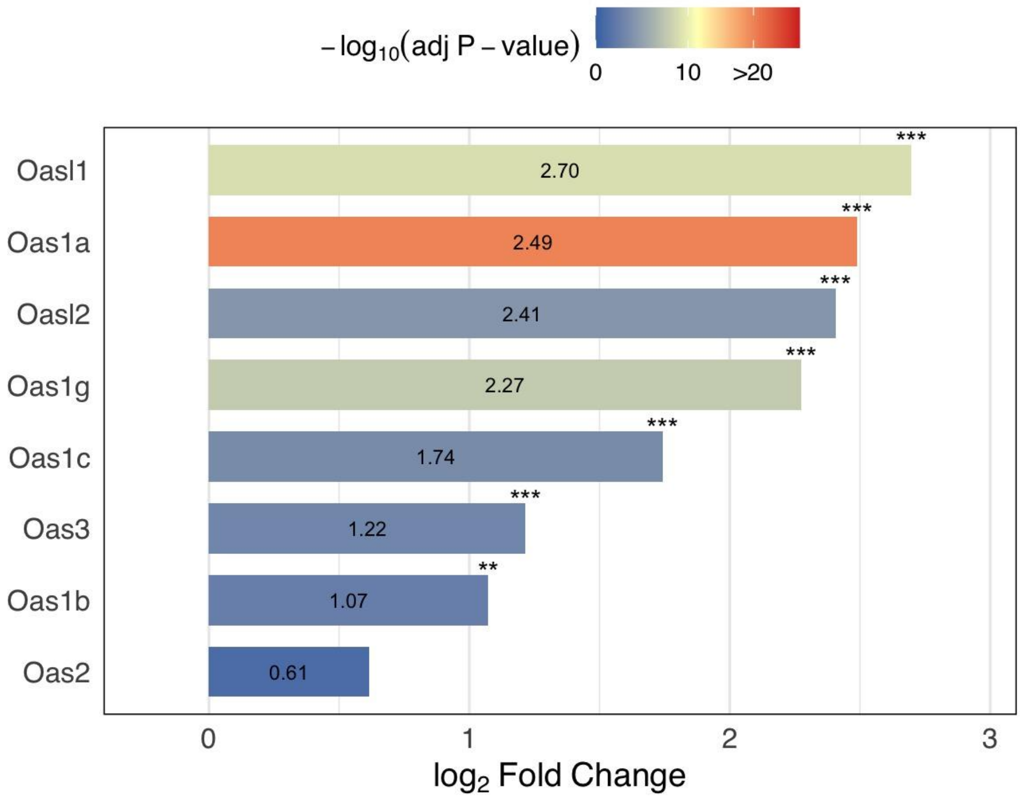

Supplementary Figure: Differential Gene Expression Analysis of OAS Genes

This bar plot illustrates the log_2 fold change in expression levels of eight OAS genes. Each bar represents the magnitude of gene expression changes, with the log_2 fold change values displayed inside the bars. The significance of differential expression is indicated by stars positioned outside the bars: ***$p < 0.001$, **$p < 0.01$, *$p < 0.05$.
